# Age differences in medial prefrontal activity for subsequent memory of truth value

**DOI:** 10.3389/fpsyg.2014.00087

**Published:** 2014-02-07

**Authors:** Brittany S. Cassidy, Trey Hedden, Carolyn Yoon, Angela H. Gutchess

**Affiliations:** ^1^Department of Psychology, Brandeis UniversityWaltham, MA, USA; ^2^Athinoula A. Martinos Center for Biomedical Imaging, Massachusetts General HospitalBoston, MA, USA; ^3^University of MichiganAnn Arbor, MI, USA

**Keywords:** medial prefrontal cortex, truth value, aging, encoding, insula

## Abstract

Much research has demonstrated that aging is marked by decreased source memory relative to young adults, yet a smaller body of work has demonstrated that increasing the socioemotional content of source information may be one way to reduce age-related performance differences. Although dorsomedial prefrontal cortex (dmPFC) activity may support source memory among young and older adults, the extent to which one activates dorsal vs. ventral mPFC may reflect one's personal connection with incoming information. Because truth value may be one salient marker that impacts one's connection with information and allocation of attention toward incoming material, we investigated whether the perceived truth value of information differently impacts differences in mPFC activity associated with encoding source information, particularly with age. Twelve young (18–23 years) and 12 older adults (63–80 years) encoded true and false statements. Behavioral results showed similar memory performance between the age groups. With respect to neural activity associated with subsequent memory, young adults, relative to older adults, exhibited greater activity in dmPFC while older adults displayed enhanced ventromedial prefrontal cortex (vmPFC) and insula engagement relative to young. These results may potentially indicate that young adults focus on a general knowledge acquisition goal, while older adults focus on emotionally relevant aspects of the material. The findings demonstrate that age-related differences in recruitment of mPFC associated with encoding source information may in some circumstances underlie age-equivalent behavioral performance.

## Introduction

In addition to remembering the information itself, some of the most important choices in life require consideration of the context related to incoming information, often referred to as source memory (Johnson and Raye, 1981; Johnson et al., 1993). For example, the reliability of a source may be crucial when evaluating health claims (e.g., should you believe an endorsement that a dietary supplement can increase longevity if the endorser helped develop the supplement?). A small body of behavioral evidence shows that increasing task salience, or the degree of importance of a task to an individual, may reduce age differences in source memory (e.g., Rahhal et al., 2002; but see Siedlecki et al., 2005), in contrast with the age-related source memory impairments shown for tasks across several domains of stimuli that utilize less salient (e.g., focusing on perceptual information, as in Rahhal et al., 2002) and non-social material (e.g., Spencer and Raz, 1995). This suggests that for salient information (e.g., using affective or value-based information, as in Rahhal et al., 2002), older adults may not show the same pattern of age-related impairment that is typically found in tasks involving less salient information.

Purported truth might be one salient factor driving prioritization of information at encoding, and may reduce age differences in source memory, given the inherent value of knowing whether information is true. For instance, while both the *National Enquirer* and *The New York Times* are sources of information, people may perceive non-tabloid journalism as more truthful, and more deeply encode this information. These decisions occur in the interpersonal realm as well. You might, for example, learn the same piece of information from a gossiping co-worker known to spread rumors, or from a trusted friend; you may allocate more attention to your friend due to the perceived reliability of that source. This prioritization in encoding strategies suggests that source memory, or remembering the context in which information was presented, may directly relate to how we process information obtained from our daily interactions.

It is possible that the neural activity supporting age-equivalent source memory for socially salient material involves the interaction of cortical systems with the medial temporal lobe (MTL) that are distinct from the cortical-MTL interactions underlying non-social sources, which undergo age-related decline. Processing socially meaningful vs. non-social information recruits a reliable network of brain regions (Mitchell et al., 2002, 2005), and the engagement of medial prefrontal cortex (mPFC) in particular has been documented in a range of tasks involving socioemotional processing (Van Overwalle, 2009), including memory for information encoded during a social vs. a non-social orienting task (e.g., Mitchell et al., 2004).

Work exploring potential age differences in mPFC function during social tasks shows evidence for equivalent engagement for young and older adults (Gutchess et al., 2007; Beadle et al., 2012; Cassidy et al., 2012) as well as evidence for decreased activity with age (Moran et al., 2012). The diverging patterns of age effects on dorsomedial prefrontal cortex (dmPFC), however, may reflect when information is personally salient (Beadle et al., 2012; Cassidy et al., 2012) vs. when it is not (Moran et al., 2012). For instance, for the socially salient task involving self-referencing, young and older adults similarly recruit dmPFC for the processing of self- vs. other-related information (Gutchess et al., 2007) and for successful encoding of source materials presented in a self- vs. other-referential way (Leshikar and Duarte, 2013). However, older adults may not activate dmPFC to the level of young (e.g., Moran et al., 2012) unless a task explicitly involves socially and personally salient information.

Age-equivalent mPFC activity in personally salient social tasks may be concordant with behavioral data showing that socioemotional or value-rich materials reduce age-related performance differences (Fung and Carstensen, 2003; Carstensen and Mikels, 2005; Mather and Carstensen, 2005; May et al., 2005; Cassidy and Gutchess, 2012). Because older adults place higher priorities than young adults on value-rich, affective information (Fredrickson and Carstensen, 1990), they may perform as well as young adults on source memory tasks with value-rich material. For instance, although young adults recall perceptual and conceptual source information better than older adults, this age difference disappears when using sources relevant to emotional information (May et al., 2005) or truth value (Rahhal et al., 2002).

Recruitment of dmPFC for social tasks may also depend on one's orientation to the task, regardless of age. For instance, young adults show a dissociation in mPFC activity when thinking about similar or dissimilar others, such that dmPFC is engaged when abstractly thinking about a dissimilar other, while ventromedial prefrontal cortex (vmPFC) is recruited for thinking about self-similar others and emotional states (Mitchell et al., 2006a). This raises the possibility that age differences in mPFC activity may in part be due to differences in how young and older adults naturally orient to social tasks (e.g., they approach the learning of social information in an abstract way largely devoid of personal involvement, or with more of an emotional orientation). Supporting the idea of task orientation influencing mPFC activity, recent work has demonstrated that age differences in mPFC engagement toward processing valenced social material are driven by dmPFC in young adults, and by vmPFC in older adults (Cassidy et al., 2013). It is unknown, however, whether young and older adults display overall differences in neural activity, and specifically dissociations in dorsal and ventral mPFC activity, for salient tasks that are not overtly positive or negative.

A tendency to focus on value-rich and emotional information with age is reflective of Socioemotional Selectivity Theory, which posits that older adults tend to focus on processing information with enhanced value and socioemotional meaning due to the feeling that time in one's life is limited (Carstensen et al., 1999). In contrast, young adults are proposed to have a more general focus on acquiring knowledge. Illustrating this concept, older adults have been shown to remember more advertisements featuring emotionally meaningful messages, whereas young adults do not display a bias based on the type of presented material (Fung and Carstensen, 2003). If older adults approach tasks involving value-rich stimuli with an emotional orientation, then more vmPFC activity rather than dmPFC activity might be expected in older vs. young for such tasks. This is because ventral subregions of mPFC have been implicated in affective processing (Davidson and Irwin, 1999), including subsequent memory for emotional material (Dolcos et al., 2004), and for affective components, as opposed to cognitive components, in theory of mind tasks (Shamay-Tsoory and Aharon-Peretz, 2007). In contrast, dmPFC has been implicated in more complex cognitive operations (e.g., see Saxe, 2006).

Another distinction in dorsal and ventral mPFC activity involves the extent of their engagement during controlled and automatic processing tasks (Satpute and Lieberman, 2006; Lieberman, 2007). For instance, automatic processing has been likened to reflexive processing and with vmPFC activity during the formation of intuitions (Bechara et al., 1997). In contrast, controlled processing has been likened to reflective processing, and has been linked with increased dmPFC activity when using reappraisal to reduce emotional response to negative scenes (Ochsner et al., 2002). Older adults have larger memory deficits on tasks involving controlled processing, relative to automatic processing (Hasher and Zacks, 1979), perhaps because many tasks do not motivate older adults to access controlled processing resources (Germain and Hess, 2007). When motivated by socioemotional goals, however, older adults may be more likely to allocate the cognitive resources necessary for controlled processing toward their goal, as in the case of emotion regulation (Carstensen et al., 2003). Thus, in the case of a context where information is value-rich, but the task itself is not necessarily emotionally meaningful, we might expect young, but not older, adults to use controlled processing to encode information even if they are not instructed to do so, in the service of knowledge acquisition goals. In contrast, older adults may employ automatic processing during similar tasks. For a task involving value-rich stimuli, this might be reflected in enhanced vmPFC activity among older adults relative to young adults.

The present study explored whether young and older adults differentially engage dmPFC and vmPFC for the value-rich task of encoding truth value, be it inferred from a social source or explicitly stated. Our task involved learning the purported truth value of health-related statements, which provides a unique opportunity to appeal to the processing strategies of both young and older adults. Because health-related statements may hold particular importance for older adults, age differences in behavioral source memory for truth value may be reduced compared to the typical finding of deficits with age. Given previous findings of age differences in dmPFC and vmPFC activity as a function of the content of incoming salient information (Leclerc and Kensinger, 2008, 2010; Cassidy et al., 2013), we expected that young adults would show enhanced dmPFC activity relative to older adults for subsequent memory based on the idea that learning true and false statements might signal an inherent knowledge acquisition focus in the young. In contrast, we expected that older adults would exhibit increased vmPFC activity relative to young adults for subsequent memory, given that the more value-based aspects of health-related statements to older adults may make them more likely to approach the task with a socioemotional orientation. Given a potential socioemotional orientation among older adults, the insula, due to its broad role in emotional processing, was another neural region where increased activity might be expected in older vs. young adults for the encoding of truth value (Adolphs et al., 2000; Phan et al., 2002; Sanfey et al., 2003; Barrett et al., 2004; Craig, 2009; Wiech et al., 2010; Zaki et al., 2012). Insofar as the insula is engaged when making decisions in an emotional context (Sanfey et al., 2003), greater insula recruitment might be expected among individuals who orient to material in an emotional way (e.g., older adults) relative to those less likely to do so (e.g., young adults).

## Methods

### Participants

Twelve right-handed University of Michigan students (18–23 years old, *M* = 20.5, *SD* = 1.2, 58% female) and twelve right-handed community dwelling older adults (63–80 years old, *M* = 71.1, *SD* = 5.5, 66% female) were recruited for the study. Older adults were screened for abnormal orientation scores (<27) using the Mini-Mental State Examination (*M* = 28.7, *SD* = 1.1), and had marginally more years of education (*M* = 15.4 years, *SD* = 2.7) than young adults (*M* = 13.6 years, *SD* = 1.7), *t*_(22)_ = 1.95, *p* = 0.06. To ensure the samples' comparability with those in the prior literature, participants completed a vocabulary measure (Shipley, 1986), digit and pattern comparison tasks (Salthouse and Babcock, 1991), forward and backward digit span measures (Wechsler, 1981), and a letter-number sequencing measure (Wechsler, 1997). Results are summarized in Table 1. Participants reported no history of psychiatric or neurological disorders and no history of drug or alcohol abuse at the time of testing. The University of Michigan Institutional Review Board approved the study, and written informed consent was obtained from all participants.

**Table 1:** **Means (*SD*) for cognitive measures**.

**Measure**	**Young adults**	**Older adults**	***t***	***p*-value**
Shipley vocabulary	31.1 (3.6)	37.3 (2.9)	−4.37	<0.001
Digit comparison	28.6 (2.8)	22.2 (4.0)	4.10	0.001
Pattern comparison	24.2 (2.3)	16.2 (4.7)	4.70	<0.001
Forward digit span	8.2 (1.3)	7.9 (1.2)	0.55	0.60
Backward digit span	6.4 (1.1)	5.8 (1.7)	0.78	0.45
Letter-number sequencing	5.4 (1.0)	5.1 (0.9)	0.86	0.40

### Stimuli

The stimuli consisted of 240 true statements regarding health-related information for which participants were unlikely to have prior knowledge (e.g., “Women's hearts beat faster than men's”). Altering words in each true statement created factually untrue statements (e.g., “Men blink nearly twice as much as women”). After creating a factually untrue version of each statement, each statement was randomly assigned to one of two sets (Set A and Set B). For half of the participants, Set A consisted of true versions and Set B of false. For the other half of participants, Set A consisted of false versions and Set B of true. Thus, whether each participant saw a true or false version of any given statement was fully counterbalanced across participants. The order of statements from each set was randomly drawn for each participant. These statements have been used in prior aging research (Skurnik et al., 2005) and are available upon request. All stimuli were programmed and presented using E-Prime software (Psychological Software Tools, Pittsburgh, PA) and IFIS 9.0 (MRI Devices, Waukesha, WI).

### Truth judgment task

Participants were told they would see statements coupled with an indication of truth value, and would later be asked to remember if each statement was true or false. The task comprised a 2 (Truth Value: True/False) × 2 (Source Type: Inferred/Stated) design, in which source memory for different types of information was compared across age groups. Some statements were stated to be true or false (stated: “TRUE” or “FALSE”), without referencing a social source. Other statements were inferred to be true or false, based on the source (inferred: “PAT says:” or “CHRIS says:”). Prior to encoding, participants were introduced to two hypothetical individuals, Pat and Chris. One individual was described as being honest and trustworthy, and participants were told to assume that every statement obtained from this person was true. The other individual was described as very dishonest and untrustworthy, and participants were told to assume this person only made false statements. Half of the statements were true and the other half false, and each of these were randomly assigned to each participant for the corresponding inferred or stated source. Participants practiced the task and received feedback on their responses before completing the full encoding task in the scanner.

**Figure 1:**
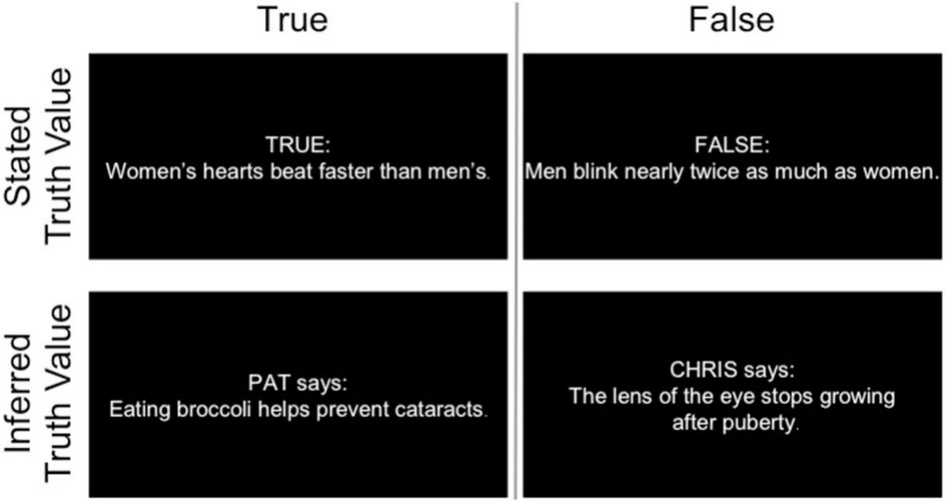
**Examples of encoding stimuli used in each condition**.

Statements and truth value indications were presented on the screen concurrently for 6 s (see Figure 1). Participants selected whether presented information was true or false via button press. Responses were monitored during scanning to ensure that participants were accurately reporting the truth status of statements. No participant exhibited difficulty with this aspect of the task. Each run contained 60 statements, pseudorandomly distributed among baseline periods of fixation ranging from 2 to 12 s. Condition order and baseline periods were determined using Optseq2 (http://surfer.nmr.mgh.harvard.edu/optseq).

Participants completed the source memory task outside the scanner approximately 15–20 min after structural scans and four encoding runs. Participants were reminded of the truth values associated with each person (e.g., “If you remember that Pat said it, it must be true; if Chris said it, it must be false.”), were informed that they had seen all statements previously in the scanner, and were told that half the statements were true and half false. During the source memory task, participants saw each of the 240 statements displayed during encoding, with no indication of truth value. Participants indicated whether they were confident a statement was true, guessing that it was true, guessing it was false, or confident it was false, via a four-choice button press.

### Image acquisition

Data were acquired using a 3T GE LX MR scanner (GE Signa 9.0 VH3 software, General Electric, Milwaukee, WI) paired with a whole-head coil. Two hundred and seventy volumes were obtained in each of four functional runs, using a gradient-echo spiral acquisition sequence to measure blood oxygen level-dependent (BOLD) effects (*TR* = 2000 ms, *TE* = 25 ms, flip angle = 80, 64 × 64 matrix, FOV = 200 mm). Dummy scans were also acquired, but discarded for analyses. Thirty-two contiguous oblique slices were acquired parallel to the AC/PC line. T1 Structural images were acquired using a Spoiled GRASS sequence (FOV = 24 mm, 256 × 192 matrix), of 120 sagittal slices (0.9373 mm in-plane resolution) of 1.5 mm thickness.

### Data analysis

#### Behavioral data

Behavioral data were analyzed using an alpha level of 0.05. Responses to a statement involving a confident correct truth value attribution were classified as correct source memory for that statement. Guessing responses and incorrect attributions were classified as incorrect source memory for that statement, in keeping with prior research (Otten et al., 2001).

#### fMRI data

Functional volumes were slice time corrected with an 8-point Hanning windowed sinc interpolation implemented in C++. Subject motion correction was performed using AIR 3.08 (Woods et al., 1992). Remaining analyses were performed using SPM2 (Wellcome Department of Cognitive Neurology, London, UK) and programs from the Gablab Toolbox (Massachusetts Institute of Technology, Cambridge, MA). The anatomical image was coregistered to the fifth functional volume and normalized to MNI space using a standard T1 template image with 2 mm^3^ voxels. Normalization parameters determined from the anatomy were applied to functional volumes, which were then smoothed with a 6 mm isotropic Gaussian kernel. Effects for the eight stimulus conditions [i.e., 2 (Truth Value: True/False) × 2 (Source Type: Inferred/Stated) × 2 (Subsequent Memory: Correct/Incorrect)] were estimated using event-related regressors convolved with a canonical hemodynamic response function.

Results reported are from group-level, random effects analyses using an uncorrected threshold of *p* = 0.005 with a cluster size of *k*>11, chosen to ensure that activations spanned at least two acquired contiguous voxels. Although we used a fairly liberal threshold, we took a whole-brain rather than a region of interest approach because relatively little prior work on this topic made it desirable to test a wider array of regions showing age-related changes in subsequent source memory in order to inform future work. Functional images from encoding were back-sorted for further analyses according to whether truth value was subsequently remembered (correct: high confidence correct responses only) or forgotten (incorrect: summing across guess responses and confident incorrect attributions). Subsequent memory contrasts (young > old, correct > incorrect source memory; old > young, correct > incorrect source memory) were used to initially analyze functional data and to identify regions demonstrating age differences where correct source memory produced increased engagement over incorrect source memory. The correct > incorrect source memory contrast was defined at the subject-level individually, and then entered in a group-level analysis for simple one-way group-wise differences. Locations of peak activation on the cortical surface were identified using SPM2, and Brodmann areas were obtained with MRIcron (Rorden and Brett, 2000). To characterize age differences in subsequent source memory from our whole-brain analysis, parameter estimates for each condition from each participant were extracted from the peak voxels of our a priori regions of interest (dmPFC, vmPFC, and insula) where significant activations emerged in the correct > incorrect source memory contrast.

## Results

### Behavioral results

Proportions of recognition responses in each category (confident true, guessing true, guessing false, confident false) are shown in Table 2. From these responses, two behavioral measures were computed to assess memory performance. First, the proportion of correct source memory judgments (confident correct attributions; e.g., confident true when the statement was true) was calculated. Second, the false alarm rate was calculated as the proportion of statements incorrectly assigned a confident attribution (e.g., confident true when the statement was false). We analyzed these measures separately as dependent variables using a 2 (Age Group: young, old) × 2 (Source Type: inferred, stated) × 2 (Truth Value: true, false) mixed ANOVA.

**Table 2:** **Behavioral source memory performance [*M*, (*SD*)] for young (YA) and older (OA) adults**.

	**Confident true**		**Guessing true**		**Guessing false**		**Confident false**	
	**True**	**False**	**True**	**False**	**True**	**False**	**True**	**False**
**SOCIAL SOURCE**								
YA	0.70 (0.19)	0.13 (0.06)	0.15 (0.14)	0.12 (0.11)	0.10 (0.06)	0.19 (0.09)	0.06 (0.03)	0.56 (0.16)
OA	0.67 (0.16)	0.13 (0.10)	0.15 (0.12)	0.12 (0.11)	0.09 (0.06)	0.21 (0.14)	0.09 (0.07)	0.53 (0.25)
**NON-SOCIAL SOURCE**								
YA	0.73 (0.22)	0.13 (0.08)	0.14 (0.12)	0.14 (0.11)	0.07 (0.09)	0.20 (0.13)	0.06 (0.07)	0.54 (0.18)
OA	0.73 (0.16)	0.15 (0.12)	0.13 (0.11)	0.13 (0.11)	0.08 (0.05)	0.23 (0.12)	0.06 (0.06)	0.50 (0.22)

Correct source memory is the same as confident responses for the correct truth value (e.g., confident true for true statements). Incorrect source memory is the sum of guessing (true and false) and confident incorrect source attributions. No age group differences were significant in any condition.

For correct source memory judgments, there was a main effect of Truth Value, *F*_(1, 22)_ = 45.84, *p* < 0.001, η^2^_*p*_ = 0.68, such that participants correctly identified more true (*M* = 0.71, *SD* = 0.18) than false (*M* = 0.53, *SD* = 0.20) statements. There were no main effects of Age Group or Source Type. There was also a significant interaction between Source Type and Truth Value, *F*_(1, 22)_ = 9.42, *p* < 0.01, η^2^_*p*_ = 0.30. Contrasts showed that true statements coming from a stated source (*M* = 0.73, *SD* = 0.19) were better remembered than true statements coming from a inferred source (*M* = 0.68, *SD* = 0.17), *F*_(1, 22)_ = 7.69, *p* < 0.01, η^2^_*p*_ = 0.26. In contrast, accuracy in identifying false statements from inferred (*M* = 0.54, *SD* = 0.20) and stated (*M* = 0.52, *SD* = 0.20) sources did not differ, *F*_(1, 22)_ = 1.33, *p* = 0.26, η^2^_*p*_ = 0.06. No other interactions approached significance.

For false alarm rates, there was a main effect of Truth Value, *F*_(1, 22)_ = 15.46, *p* < 0.001, η^2^_*p*_ = 0.41. Participants were more likely to confidently remember an originally false statement as true than an originally true statement as false. No other effects approached significance.

The absence of behavioral interactions involving Source Type and Age Group suggests that source memory in aging does not depend on whether information was inferred via a person's reliability or explicitly stated. However, prior fMRI results have suggested that social information processing is neurally distinct from processing other types of information (Mitchell et al., 2002, 2004; Van Overwalle, 2009). We accordingly characterized our imaging data to determine if the processing mechanisms involved in encoding inferred vs. stated truth value were similarly distinct, and how aging might modulate the engagement of these regions.

### fMRI results

We identified brain regions showing age differences in response to overall subsequent memory, given the relatively low number of participants in this study (*N*s = 12 for each age group) (correct > incorrect memory; see Table 3). This contrast captures regions that differ across age groups, but is conservative with respect to the source and truth of each statement. Parameter estimates from the peak voxel of these functionally defined regions were probed to characterize whether age differences in activity was driven by Source Type or Truth Value (Figure 1). However, specific contrasts involving these factors were not conducted due to low power and the lack of behavioral evidence for source memory differences with age depending on Source Type or Truth Value.

**Table 3:** **Age differences in brain activity for correct > incorrect source memory**.

**Region**	***BA***	***k***	**Activation Peak *(x*, *y*, *z)***			***t***
**(A) YOUNG > OLD, CORRECT > INCORRECT SOURCE MEMORY**						
L dorsomedial prefrontal cortex	6/8	68	−14	32	54	5.70
L dorsomedial prefrontal cortex	6	33	−14	12	66	3.60
R amygdala		28	20	−2	−24	3.87
R superior frontal gyrus	6	14	14	−16	78	3.52
L precuneus	19	15	−30	−72	32	3.25
L inferior frontal gyrus	9	14	−52	20	24	3.08
**(B) OLD > YOUNG, CORRECT > INCORRECT SOURCE MEMORY**						
L ventromedial prefrontal cortex	10/11	66	−16	56	−2	4.01
L medial prefrontal cortex	10		−8	52	−4	3.16
L anterior cingulate gyrus	32		−14	46	−4	3.08
R insula		37	44	−6	−2	3.26
R precuneus	7	224	16	−66	52	5.81
L dorsolateral prefrontal cortex	9/46	75	−40	36	36	5.36
L middle frontal gyrus	46		−46	36	24	4.10
L precentral gyrus	4	50	−52	−16	28	4.69
R middle frontal gyrus	8	180	26	36	40	4.69
R cingulate gyrus	32		18	22	36	3.43
L cingulate gyrus	31	25	−20	−28	40	4.48
L insula		12	−28	−22	24	4.08
R inferior parietal lobule	40	64	68	−26	26	3.77
R superior parietal lobe	5	40	20	−52	58	3.66
R thalamus		19	10	−38	10	3.59
R middle frontal gyrus	6	13	26	4	42	3.58
L inferior parietal lobule	40	20	−34	−42	48	3.57
R inferior frontal gyrus	47	27	36	36	2	3.56
L superior parietal lobule	5	12	−22	−46	68	3.53
R posterior cingulate gyrus	29	18	6	−50	4	3.53
R superior frontal gyrus	6	20	8	−10	70	3.52
R insula		15	38	22	6	3.45
R inferior temporal gyrus	20	24	50	−34	−14	3.41
R fusiform gyrus	37	12	−12	58	20	3.26
L postcentral gyrus	40	15	−54	−22	14	3.26
R middle frontal gyrus	10	28	32	56	22	3.24
R superior frontal gyrus	10		22	62	24	3.05
R superior frontal gyrus	9	11	10	56	30	3.06

The data show regions emerging in the contrast with an overall threshold of p < 0.005 and an extent threshold of 11 voxels. Regions listed without a cluster size are subsumed by the larger cluster listed directly above. A priori regions of interest are listed first. Other regions are listed from highest to lowest t-value. L, Left; R, right; k, cluster size.

In a whole-brain analysis, this subsequent memory contrast yielded brain regions contributing to age differences in correct subsequent memory for source (Table 2). To probe the expected age differences related to socioemotional processing in the dmPFC, vmPFC, and insula, we focused on these regions. Parameter estimates of activation attributable to each condition were then extracted and compared in 2 (Age Group: young, old) × 2 (Source Type: inferred, stated) × 2 (Truth Value: true, false) mixed ANOVAs.

**Figure 2:**
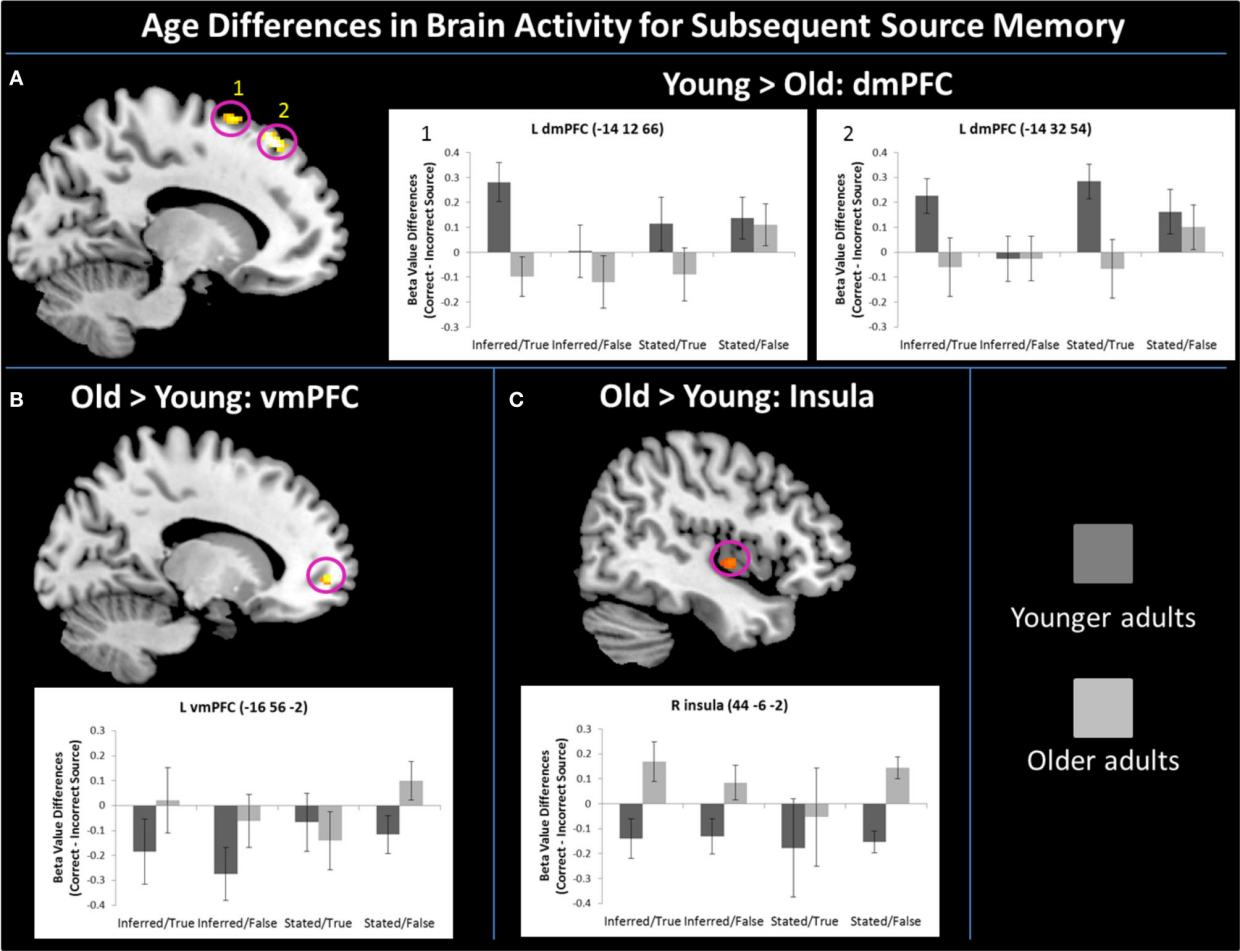
**Age differences for correct—incorrect source memory for truth value existed in mPFC, such that young adults had enhanced activity over older adults in two regions of left dmPFC (A), while older adults had increased recruitment over young in left vmPFC (B).** Older adults also had enhanced activity relative to young adults for subsequent memory of truth value in the right insula **(C)**. Error bars represent standard error of the mean.

As defined by the contrast, young adults had increased activation compared to older adults in two regions of left dmPFC [Figure 2A; BA 6: *F*_(1, 22)_ = 9.43, *p* < 0.01, η^2^_*p*_ = 0.30; BA 8: *F*_(1, 22)_ = 16.48, *p* < 0.001, η^2^_*p*_ = 0.43]. Characterizing this effect further (and probing effects unrelated to the region-defining contrast), there were marginal Age Group by Truth Value interactions in both regions of dmPFC [BA 6: *F*_(1, 22)_ = 3.01, *p* = 0.097, η^2^_*p*_ = 0.12; BA 8: *F*_(1, 22)_ = 3.43, *p* = 0.08, η^2^_*p*_ = 0.14]. Pairwise comparisons showed that in both dmPFC regions, this interaction appeared to be driven by enhanced activity in young relative to older adults when successfully encoding true (BA6: *p* = 0.002, BA 8: *p* = 0.004) vs. false (BA 6: *p* = 0.40, BA 8: *p* = 0.70) statements.

As defined by the contrast, older adults had increased vmPFC engagement compared to young adults in a region of left vmPFC [Figure 2B; *F*_(1, 22)_ = 3.43, *p* = 0.002, η^2^_*p*_ = 0.35]. Examination of parameter estimates for effects unrelated to the region-defining contrast showed that unlike the marginal Age Group by Truth Value interactions in dmPFC activity, vmPFC showed a marginal Age Group by Source Type interaction, *F*_(1, 22)_ = 3.46, *p* = 0.08, η^2^_*p*_ = 0.14. Young adults engaged vmPFC more for the successful encoding of truth value from stated vs. socially-inferred sources, *F*_(1, 11)_ = 4.35, *p* = 0.06, η^2^_*p*_ = 0.28, while older adults, showed no such difference, *F* < 1, *p* = 0.93. There was also a marginal main effect of Source Type, such that all participants tended to recruit vmPFC for the successful encoding of stated vs. inferred truth value, *F*_(1, 22)_ = 3.11, *p* = 0.09, η^2^_*p*_ = 0.12. Finally, older adults had increased neural activity relative to young adults (as defined by the contrast) in the right insula (Figure 2C), *F*_(1, 22)_ = 9.55, *p* < 0.01, η^2^_*p*_ = 0.30, for the successful encoding of truth value. Unlike the marginal Truth Value by Age Group interactions in dmPFC and Source Type by Age Group interaction exhibited in vmPFC during the task, no interactions were present in insula recruitment.

## Discussion

Previous research on source memory and aging has detailed decreases in older adults' behavioral performance (Chalfonte and Johnson, 1996), and neural activity (Mitchell et al., 2006b; Dennis et al., 2008) when compared to a young cohort, indicating overall age-related decline in encoding source information. However, a smaller body of behavioral work suggests that increasing the salience of stimuli can reduce age-related source memory impairments (Rahhal et al., 2002; May et al., 2005), and that older adults' memory may be enhanced when information is initially encoded in a salient way (Cassidy and Gutchess, 2012). The current study extended this idea by asking if the value-rich task of encoding truth value would be represented by differential activation of mPFC with age, despite potential age-equivalent memory performance. If young adults rely more on knowledge acquisition goals when learning salient information, greater activity in *dorsal* mPFC would be expected, whereas greater activity in *ventral* mPFC and in the insula would be expected for older adults, representing an approach to the task that reflects more socioemotional processing. Our findings are consistent with these expectations, suggesting that in these circumstances, age-equivalent performance may be supported by differential neural recruitment.

The demonstrated dorsal-ventral shift in encoding-related activity with age could also be reflective of overall changes in processing focus, as posited by Socioemotional Selectivity Theory (Carstensen et al., 1999). This theory puts forth the idea that with increasing age, people shift from a focus on acquiring knowledge to a focus on enhancing the emotional meaning in one's life. Thus, a changing focus in older adults toward material with enhanced emotional meaning might be reflected in enhanced activity in regions supporting affective processing (i.e., vmPFC) relative to young. In contrast, regions implicated in the encoding of value-rich information (i.e., dmPFC) may be more active in young than older adults, as young adults may try to optimize their likelihood of acquiring knowledge.

The current findings revealed enhanced overall dmPFC activity in young relative to older adults. While older adults have shown dmPFC activity similar to young adults during socially self-relevant processing (Gutchess et al., 2007; Beadle et al., 2012; Cassidy et al., 2012), age-related decreases have been observed in tasks involving social, but not-explicitly self-related, material (Moran et al., 2012). Given that the current task did not place older adults within such a social task, we might not expect them to activate dmPFC to the same extent as young. Increased dmPFC processing among young compared to older adults may also indicate the use of relatively more controlled processing to encode information. Additional research is needed to clarify this possibility.

Exploratory analyses provided further insight into whether stated or inferred truth value, or the truth value of information itself, drove age differences in dmPFC activity. An exploratory characterization of the greater activity in dmPFC among young adults revealed a trend toward increased activity among young over older adults during encoding of veridical, but not false, information regardless of the source. Albeit speculative, such an interaction might be expected for this task because being told that information is true might trigger allocation of attention for prioritized encoding. Enhanced dmPFC activity in young over older adults, especially for encoding true statements, may therefore reflect the overall knowledge acquisition focus among young adults posited by Socioemotional Selectivity Theory (Carstensen et al., 1999).

Older adults, in contrast, tend toward an enhanced focus on salient and emotionally meaningful material (Fredrickson and Carstensen, 1990; Carstensen and Turk-Charles, 1994; Fung et al., 1999; Carstensen et al., 2000; Carstensen and Mikels, 2005), which we suggest may be reflected in their recruitment of vmPFC and insula for subsequent memory relative to young adults. If older adults approach a task involving salient material with a focus, conscious or not, on the personally relevant or emotional aspects of the presented information, then we might expect increased engagement of vmPFC (e.g., Mitchell et al., 2006a). Previous work with young adults has shown that vmPFC is more active for tasks involving more self-similar, and thereby potentially more personally salient, information, vs. self-dissimilar social material, and work in aging has also shown that age-related biases toward positively valenced social information might be primarily reflected in vmPFC (Cassidy et al., 2013), rather than dmPFC activity. Thus, a tendency to focus on salient socioemotional material with age may result in more activation of neural regions sensitive to emotional processing during tasks that involve the encoding of value-rich material, such as truth value. Age-related increases in vmPFC engagement may also reflect the idea that, with age, enhanced focus on value-rich material may result in the recruitment of brain regions supporting more affective vs. cognitive processing.

Our findings are consistent with a growing body of work showing that for young adults (Mitchell et al., 2004; Gilron and Gutchess, 2012), and healthy older adults (Leshikar and Duarte, 2013), mPFC engagement may underlie subsequent memory for value-rich information. This is in contrast to prior literature on the encoding of non-social source material, which shows that age-related impairments in encoding source information stem from MTL dysfunction, particularly in the hippocampus (Mitchell et al., 2000; Dennis et al., 2008). Notably, we did not find age differences in hippocampal function for the task in the present study. However, our results are *consistent* with a recent study on source memory in aging that also utilized socially relevant stimuli (Leshikar and Duarte, 2013), and with work suggesting that face-trait associations are preserved in individuals with substantial hippocampal damage (Todorov and Olson, 2008). A critical follow-up to the present experiment would be to compare directly, memory for socially salient with non-social source information collected within the same task. Such a task could provide evidence that different cortical networks may interact with MTL memory systems for social vs. non-social information across age.

Importantly, the demonstrated age differences in how young and older adults recruit mPFC for encoding truth value may reflect how such information was presented to them. Although Pat and Chris were presented as sources of information, participants never learned additional behavioral information about Pat and Chris aside from learning that one was more trustworthy than the other. Speculatively, in aging, explicit person representations and comparisons may drive dmPFC activity in socially salient tasks. It could be that a lack of explicit person representation involved in assessing abstract factual statements may explain why young adults had enhanced dmPFC activity compared to older adults for the encoding of truth value. Thus, we might expect to see interactions with age in dmPFC activity for source memory related to people, but not for more abstract material. At the same time, a focus on the emotional value of health information, something that might have increased salience with age, may have led to the demonstrated age difference in vmPFC activity seen in the current task. These ideas could be further tested in future work that directly compares neural responses to both abstract and concrete social and non-social sources.

Also consistent with age differences in socioemotional orientation to the task, older adults had increased insula activity relative to young adults. However, our exploratory analysis of parameter estimates showed that enhanced activation of the right insula was not characterized specifically by the truth value of information or by the social source. Speculatively, enhanced insula activity in older relative to young adults for subsequent memory for truth value may be indicative of more emotional initial responses to incoming information (Critchley et al., 2004; Tsakiris et al., 2007; Craig, 2009; Zaki et al., 2012) rather than treating incoming material as facts that must be acquired for the pursuit of knowledge.

Limitations of the present work include the sample sizes of the young and older adult cohorts as well as the threshold for fMRI analyses. Although exploring memory for truth value from the lens of aging is an important topic with both social and public health implications, the limited statistical power due to small sample sizes restricted the present analyses from utilizing direct contrasts of potential interactions (e.g., a direct contrast exploring brain regions that might be differentially engaged with age based on whether truth value was inferred or stated), and thus the reported marginal interactions between conditions as well as null results must be interpreted with caution. Although we investigated this in an exploratory way by characterizing our regions of interest for potential differences in activity by truth value and source type, we recognize that future work should test this in a direct manner. Nonetheless, the marginal interactions we report could be fruitful for the generation of future hypotheses, and the reported effect sizes for these interactions suggest a potentially significant effect if larger sample sizes were to be employed. Moreover, the small sample sizes used here could have masked potential age differences in our behavioral data, which in turn could have guided analyses of the imaging data. Because other studies found similar behavioral performance among young and older adults for socioemotionally salient material (e.g., May et al., 2005), it is unclear whether increasing our sample size would yield age differences in memory, particularly as our means were quite similar across the age groups.

Another potential limitation of the current work involved the different age ranges of our young (5 year span) compared to older (17 year span) adult samples. This could be problematic insofar as it could have led to more age-related variance in the older vs. young adults. This notable limitation is indicative of recruitment difficulties in aging research, as fewer older adults volunteer for fMRI studies than young. Moreover, the older adults who volunteer for fMRI studies tend to be active and highly educated, allowing for the possibility that more sedentary older adults may show different patterns of brain activation. Incorporating a wider range of ages in a lifespan sample could be helpful to address these issues in future work.

Studying how young and older adults subsequently remember truth value and its underlying neural mechanism may help to clarify a public health issue of critical importance. Older adults have increased vulnerability to fraud compared to young adults (Lormel, 2001; Telemarketing fraud against older Americans, 2007; Ruffman et al., 2012). Deficits in source memory processes may in part contribute to this enhanced vulnerability because in order to successfully navigate through one's environment, one must not only consider and remember snippets of information, but also *where* the information came from. That is, failing to remember that a “fact” came from an unreliable source may lead to suboptimal decision making. For instance, one may make a bad financial investment after being given information from an untrustworthy source, or may adopt a political opinion while forgetting to consider that a biased source was reporting the information in the first place. The observed age differences in mPFC activity in response to encoding truth value may indicate that approaching the learning of social source information with a goal of acquiring knowledge could be a strategy more typical of young adults that combats against fraud vulnerability, as opposed to encoding incoming information in a more emotional way, a strategy more typical of older adults.

In summary, the current work demonstrated that for the salient task of encoding truth value of health statements, young and older adults show differential recruitment of dmPFC and vmPFC, despite showing similar behavioral performance. It would be worthwhile for future work to test explicitly how age differences in information processing styles may be associated with age-related increases in vulnerability to fraud and deception.

### Conflict of interest statement

The authors declare that the research was conducted in the absence of any commercial or financial relationships that could be construed as a potential conflict of interest.
